# Evaluation of hemostasis parameters and the role of the oxidative damage to plasma proteins in the modulation of hemostasis in patients with nephrolithiasis before and after extracorporeal shock wave lithotripsy

**DOI:** 10.1371/journal.pone.0185157

**Published:** 2017-10-02

**Authors:** Paweł Woźniak, Bogdan Kontek, Waldemar Różański, Beata Olas

**Affiliations:** 1 2nd Department of Urology, Medical University of Łódź, Pabianicka 62, Łódź, Poland; 2 Department of General Biochemistry, Faculty of Biology and Environmental Protection, University of Łódź, Pomorska 141/3, Łódź, Poland; Kermanshah University of Medical Sciences, ISLAMIC REPUBLIC OF IRAN

## Abstract

**Purpose:**

Extracorporeal shock wave lithotripsy (ESWL) is a commonly-used method in urology, which may modulate hemostasis and may induce lipid peroxidation in patients with nephrolithiasis. However, previous studies only examine changes occurring in patients 30–240 min after ESWL. The main aim of the present study was to determine whether oxidative stress may modulate the hemostatic activity of plasma in patients with nephrolithiasis before ESWL and the day after treatment ESWL. This will be performed by measuring selected parameters of hemostasis in these patients, both before ESWL and the following day, and assessing the level of oxidative damage to plasma proteins in these patients by measuring two biomarkers.

**Methods:**

Twelve patients with nephrolithiasis and 10 healthy participants were included. The following parameters of hemostasis were measured: the activated partial thromboplastin time (APTT), prothrombin time (PT), and thrombin time (TT) of plasma, the level of fibrinogen, the level of D-dimer and blood platelet count. In addition, two selected biomarkers of oxidative stress were measured: protein carbonylation level and the number of protein thiol groups.

**Results:**

No difference was observed between patients with nephrolithiasis before and after ESWL and healthy controls with regard to PT, TT or APTT. Fibrinogen concentration and blood platelet count were lower in the nephrolithiasis patients in the period after ESWL than before ESWL. The nephrolithiasis patients demonstrated elevated D-dimer concentration after ESWL. However, although oxidative damage was observed in the plasma proteins in the nephrolithiasis patients, this was not influenced by ESWL.

**Conclusion:**

Oxidative stress may induce changes of hemostasis in patients with nephrolithiasis, both before and after ESWL. In addition, changes of hemostasis parameters such as fibrinogen, blood platelet count and D-dimer level can be observed in these patients, especially after ESWL, and this may suggest that ESWL modulates hemostasis. By having a better understanding of the influence of ESWL on hemostasis, this could lead to modifying patient care for those patients at increased risk of bleeding.

## Introduction

Hemostasis is the term given to a group of mechanisms which prevent the outflow of blood from blood vessels. It is also defined as a state of dynamic equilibrium between anti- and procoagulation reactions, which may be modulated by various factors, including oxidative stress. Many systems take part in hemostasis, including the wall of the blood vessel, the clotting process with its various factors, including fibrinogen, and the fibrinolytic and phagocyte systems [1]. Blood platelets are also very important element of hemostasis. Some papers note the presence of various hemostatic complications are observed in patients with nephrolithiasis after extracorporeal shock wave lithotripsy (ESWL) [2,3]. Moreover, other complications associated with ESWL (i.e. infection and sepsis) may exist, but ESWL is generally considered as a safe treatment. In addition, other methods (i.e. surgery and Flexible Ureterorenoscopy) for treatment of kidney stones also have effect on changes in hemostasis [4,5]. Hughes et al. [3] report changes in the levels of specific biomarkers of hemostasis, i.e. plasma fibrinogen, in patients after shock wave lithotripsy (SWL); however, the effect of SWL was measured on hemostasis parameters 30–240 minutes after SWL treatment.

It is very important that the ESWL results are dependent on several technical factors, including type of lithotripsy device, energy, and frequency of pulses, coupling of the patient to the lithotripter, location of calculus, and type of anesthesia. In addition, other factors related to the patients, stone size and density, skin to stone distance, anatomy of the excretory path, and kidney anomalies are also relevant. Moreover, ESWL treatment caused an increase in the free radical generation, and a decrease in the activity of antioxidant enzymes (i.e. superoxide dismutase and catalase) in parotid glands of ESWL-treated rats [6]. Gecit et al. [7] also observed oxidative stress in the levers and diaphragm muscles of ESWL-treated rats.

The aim of our present study was to determine whether oxidative stress may modulate the hemostatic activity of plasma in patients during ESWL (before ESWL and the day after treatment). To this end, selected hemostasis parameters were measured in patients with nephrolithiasis before ESWL and the day after treatment: the activated partial thromboplastin time (APTT), prothrombin time (PT), and thrombin time (TT) of plasma, the level of fibrinogen, the level of D-dimer and blood platelet count. In addition, the levels of two biomarkers of oxidative damage to plasma proteins, protein carbonylation and the level of thiol groups in proteins, were also determined.

## Materials and methods

### Patients and samples

The blood samples were collected from 12 patients (eight men and four women; median age = 46) with similar socio-economic backgrounds who had been referred to the 2nd Department of Urology, Medical University of Lodz, Poland, for extracorporeal shock wave lithotripsy. Treatment was given as per standardized protocol using SYSTEM Sonolith® i-move device, with the mean energy 348 kV, frequency of pulses 2 Hz and 2000 pulses per procedure. The stones that were crushed were localized in the kidney and their size were from 10 to 20 mm. Before ESWL anatomy of the kidney and excretory path for all patients were investigated by intravenous urography. No anomalies were found. No kind of anesthesia was used. Demographic data and medical history were obtained at the entry of each patient to the study. A group of ten healthy individuals (six men and four women; median age = 48) who were present in the hospital for routine health checkups and used as controls. All controls were randomly-selected, non-related men and women who had never been diagnosed with nephrolithiasis nor chronic disease and were frequency matched to the cases on age. The blood and plasma samples were taken from patients and healthy participants who were eating a balanced diet of meat and vegetables and using no antioxidant supplementation. They had not taken any medications (i.e. aspirin or any other anti-platelet drugs or anti-inflammatory agents) or addictive substances (including tobacco, alcohol, antioxidant supplementation and aspirin or any other anti-platelet drugs).

The blood samples were collected from the patients before ESWL, and one day after ESWL. The plasma samples obtained from the participants, used for measuring the biomarkers of oxidative stress, were stored at -80°C within two hours of being drawn. The protein concentration in the tested samples was calculated according to Bradford [8].

Ethical approval for this study was received from the Committee for Research on Human Subjects, Medical University of Lodz (RNN/101/13/KE).

### Markers of oxidative stress

#### Carbonyl group measurement

The detection of carbonyl groups in proteins was carried out according to Levine et al. [9] and Bartosz [10]. The carbonyl group concentration was calculated using a molar extinction coefficient (ε = 22,000 M^-1^cm^-1^), and the level of carbonyl groups was expressed as nmol carbonyl groups/mg of protein. Carbonyl content was determined by taking the SPECTROstar Nano Microplate Reader- BMG LABTECH Germany.

#### Thiol group determination

The thiol group content was measured spectrophotometrically (the SPECTROstar Nano Microplate Reader- BMG LABTECH Germany) by absorbance at 412 nm with Ellman’s reagent: 5,5’-dithio-bis-(2-nitrobenzoic acid). The thiol group concentration was calculated using a molar extinction coefficient (ε = 13,600 M^-1^cm^-1^) [10–12]. The level of thiol groups was expressed as nmol thiol groups/mg of plasma protein.

### Parameters of hemostasis

#### The measurement of prothrombin time

The PT (seconds) was determined coagulometrically (BCS XP Healthcare Diagnostics Siemens, Germany) in citrated samples.

#### The measurement of thrombin time

The TT (seconds) was determined coagulometrically (BCS XP Healthcare Diagnostics Siemens, Germany) in citrated samples.

#### The measurement of APTT

The APTT (seconds) was determined coagulometrically (BCS XP Healthcare Diagnostics Siemens, Germany) in citrated samples.

#### The measurement of blood platelet concentration

Blood platelet count was performed using an automated cell counter (Sysmex XN-2000, Sysmex, Japan) in citrated samples. The platelets were measured in units x 10^9^/l.

#### The measurement of fibrinogen

Fibrinogen (g/l) concentration (in citrated samples) was measured using an analyser (BCS XP Healthcare Diagnostics Siemens, Germany).

#### The measurement of D-dimer

D-dimer (ng/ml) concentration was determined by an analyser (BCS XP Healthcare Diagnostics Siemens, Germany) in citrated samples.

### Statistical analysis

All the values in this study were expressed as mean ± SD and median. In order to eliminate uncertain data, the Q-Dixon test was performed. The distribution of the data was tested using the Kolmogorov-Smirnov test. Since the selected hemostasis parameters and biomarkers of oxidative stress were not normally distributed, the non-parametrical Mann-Whitney U-test was used for their analysis. The reported p-values were two-sided. Probabilities were considered significant when the p-value was lower than 0.05. All analyses were completed using Statistica software.

## Results

**Fig 1:**
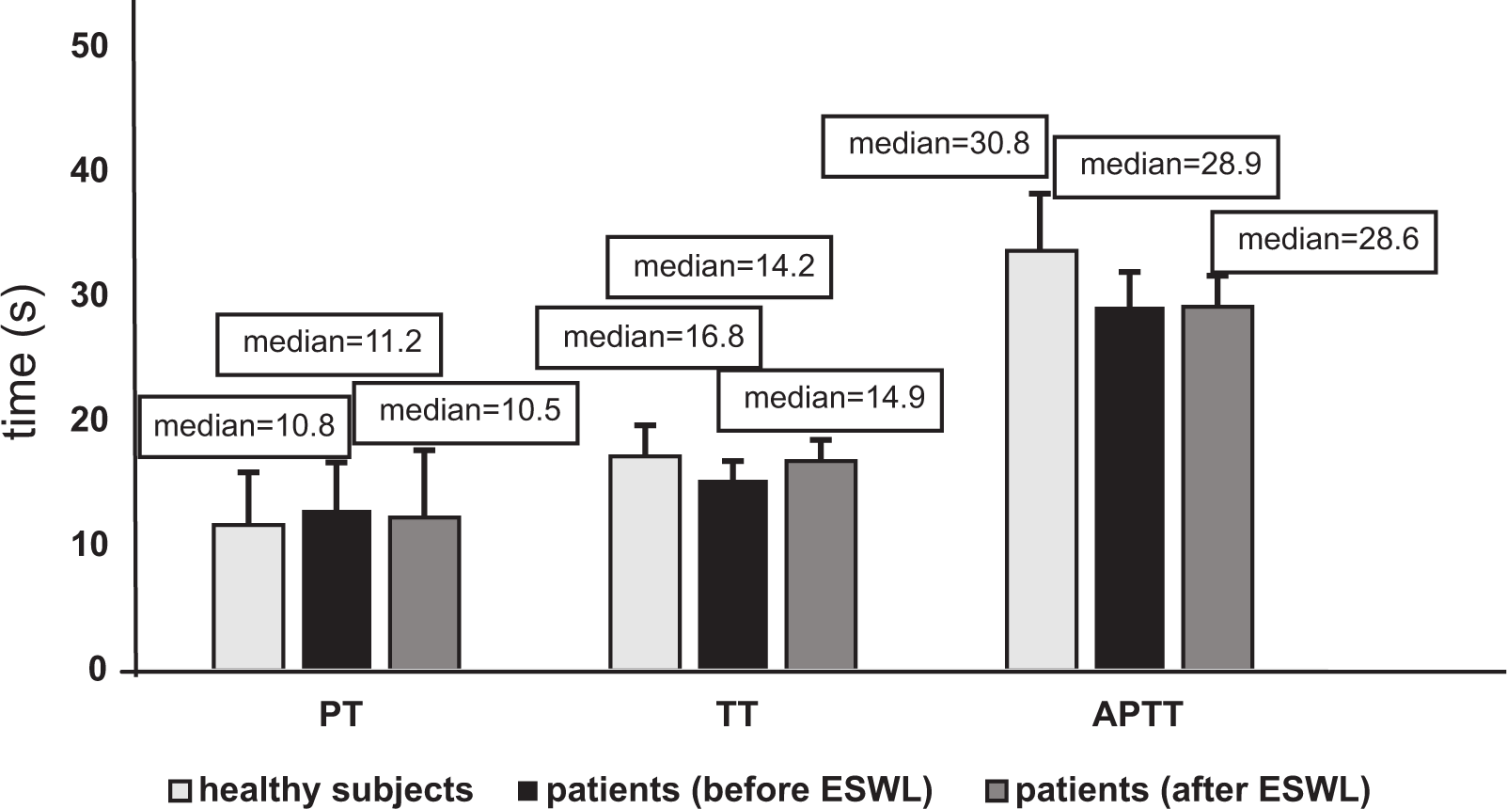
The activated partial thromboplastin time (APTT), prothrombin time (PT), and thrombin time (TT) of plasma in patients with nephrolithiasis (before and after ESWL), and in control plasma obtained from healthy volunteers. Results are given as means ± SD, and median. The statistical analysis was performed using the Mann-Whitney test (for all times: p>0.05 –patients [(before/after ESWL] *versus* healthy subjects; p>0.05 patients [after ESWL] *versus* patients [(before ESWL]).

**Fig 2:**
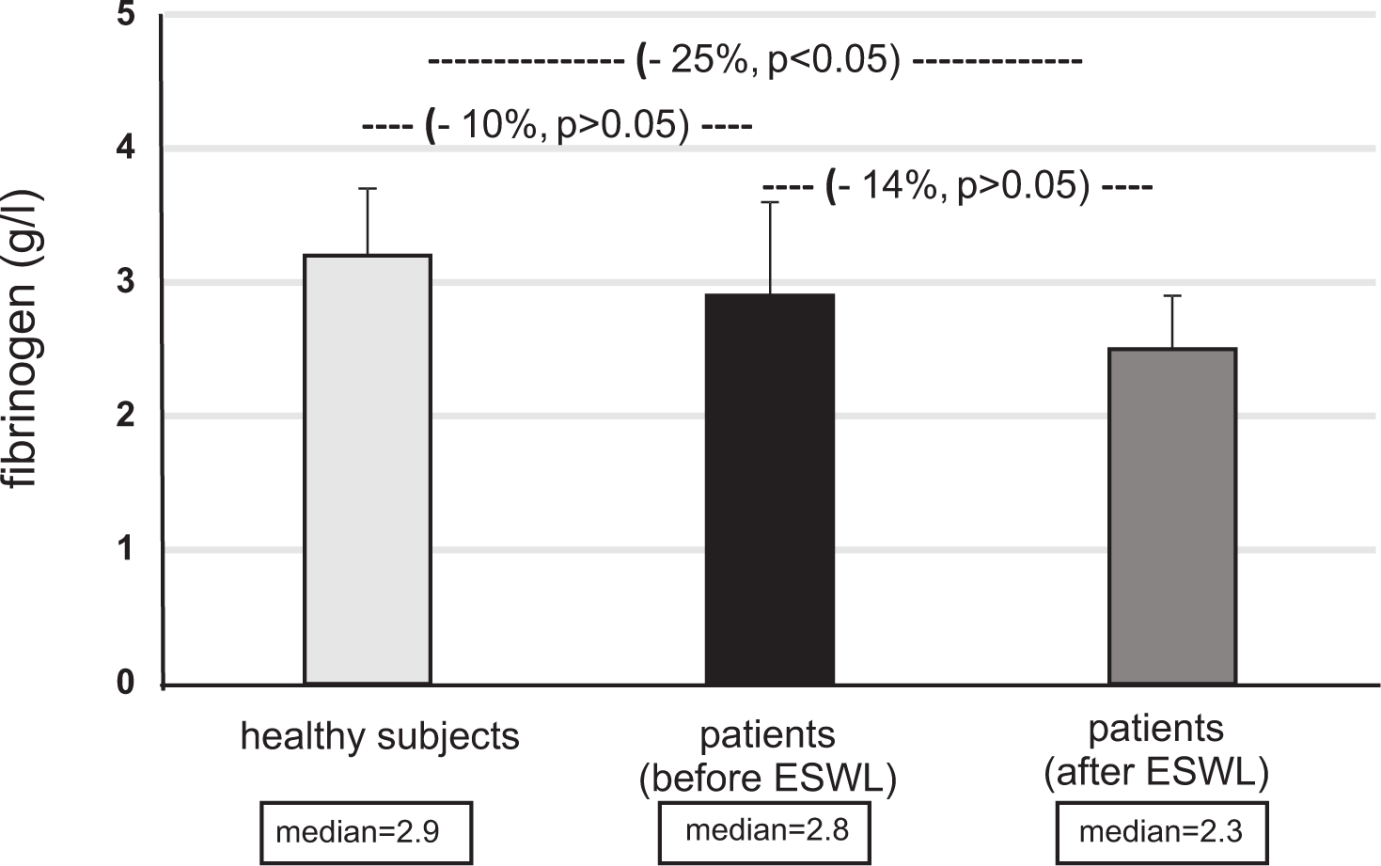
The level of fibrinogen in plasma from patients with nephrolithiasis (before and after ESWL), and in control plasma obtained from healthy volunteers. Results are given as means ± SD, and median. The statistical analysis was performed using the Mann-Whitney test.

**Fig 3:**
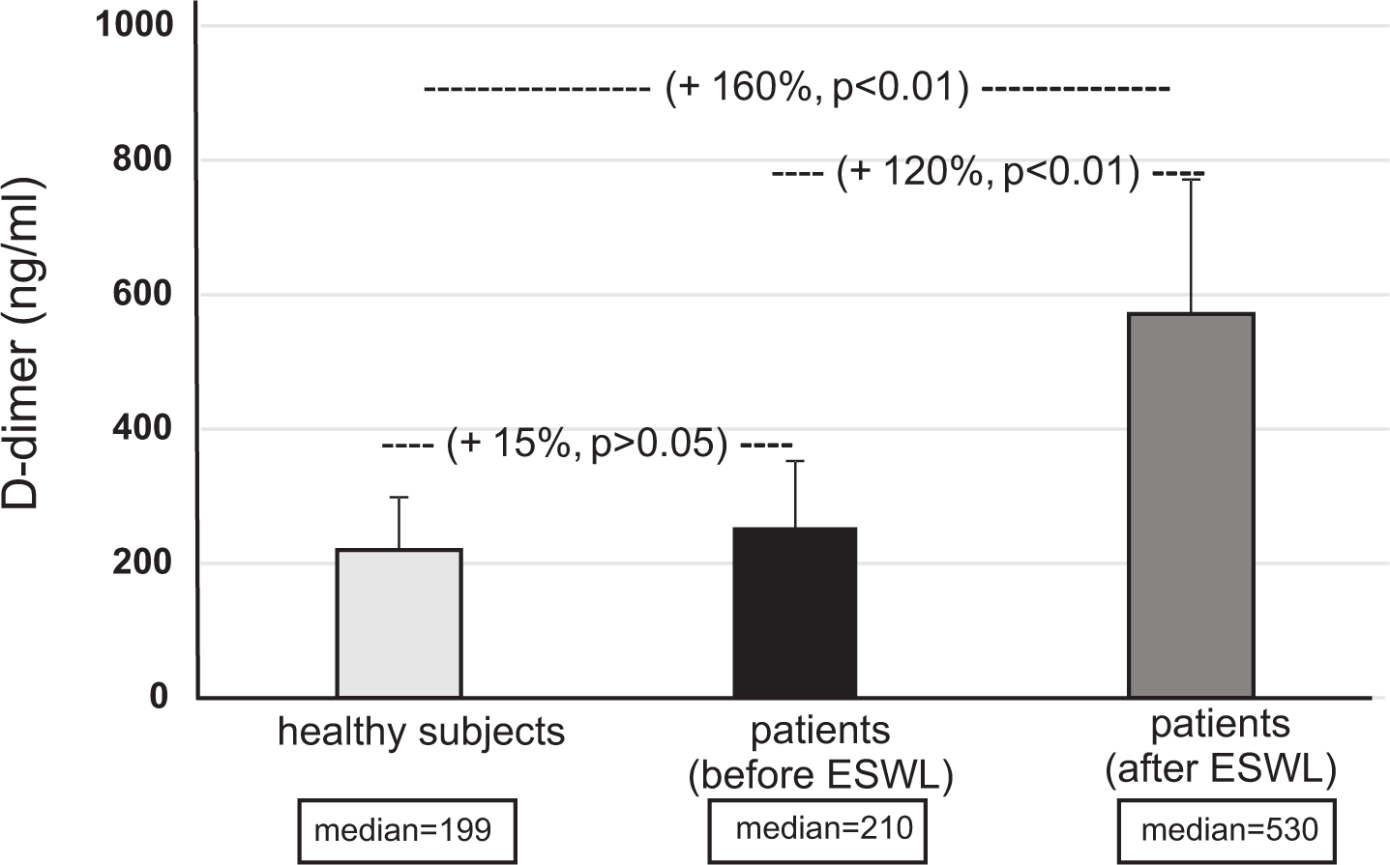
The level of D-dimer in plasma in patients with nephrolithiasis (before and after ESWL), and in control plasma obtained from healthy volunteers. Results are given as means ± SD, and median. The statistical analysis was performed using the Mann-Whitney test.

**Fig 4:**
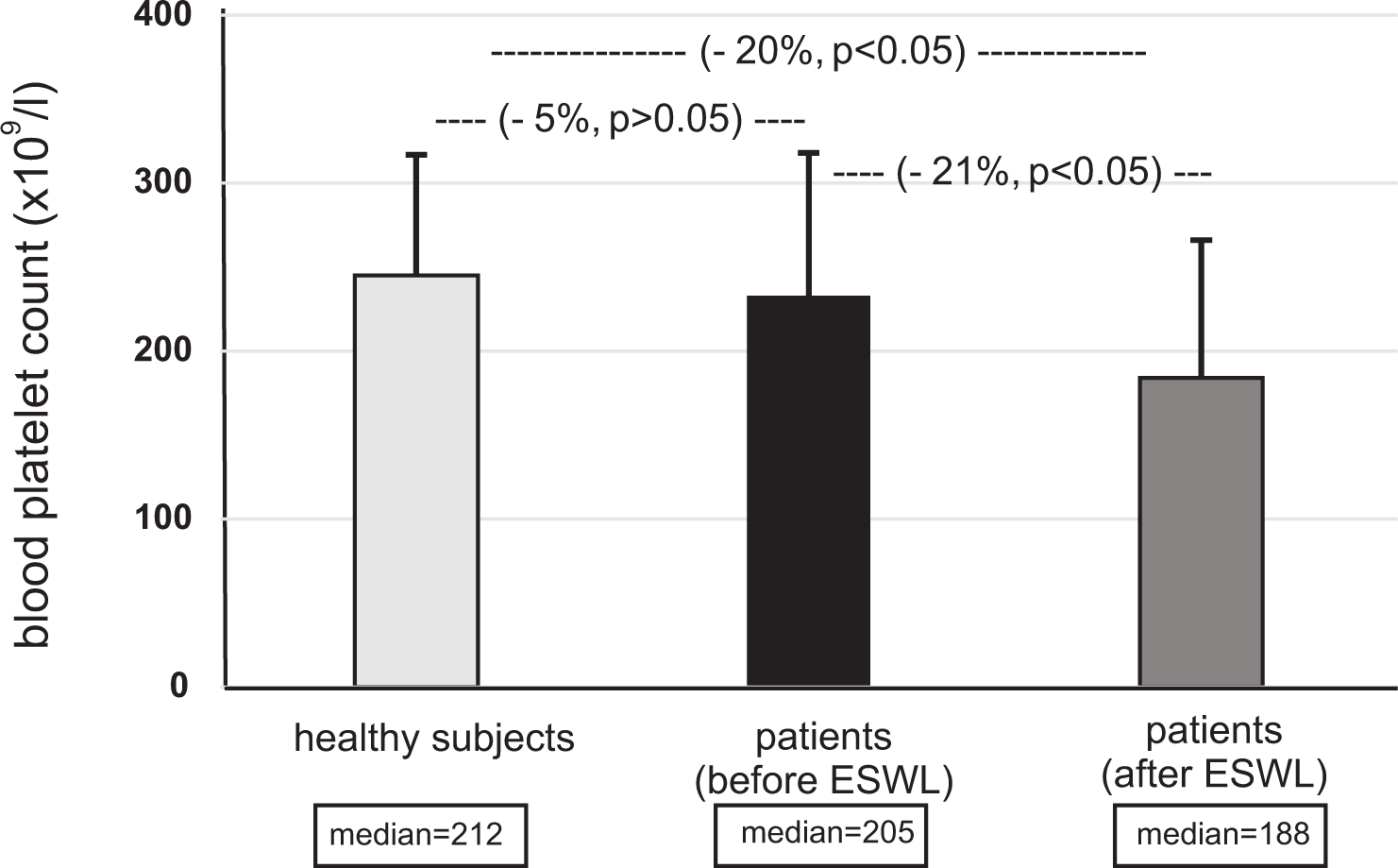
The level of blood platelets in patients with nephrolithiasis (before and after ESWL), and in healthy volunteers. Results are given as means ± SD, and median. The statistical analysis was performed using the Mann-Whitney test.

Figs 1–4 present selected parameters of hemostasis in patients with nephrolithiasis before or after ESWL. Neither APTT, PT nor TT were influenced by the presence of nephrolithiasis, nor by treatment with ESWL (p>0.05) (Fig 1, S1 Table). However, fibrinogen concentration fell, with the plasma level from the nephrolithiasis patients before ESWL reaching about 25% of control group–healthy subjects levels (p<0.05) (Fig 2). The same trend was observed for blood platelet count (p<0.05) (Fig 4). On the other hand, the concentration of D-dimer in plasma from nephrolithiasis patients after ESWL was higher than that seen in plasma obtained from healthy volunteers (p<0.01) and from patients before ESWL (p<0.01) (Fig 3). In addition, blood platelet counts were about 20% lower in patients after ESWL than in those currently before ESWL (p<0.05) (Fig 4, S2 Table).

**Fig 5:**
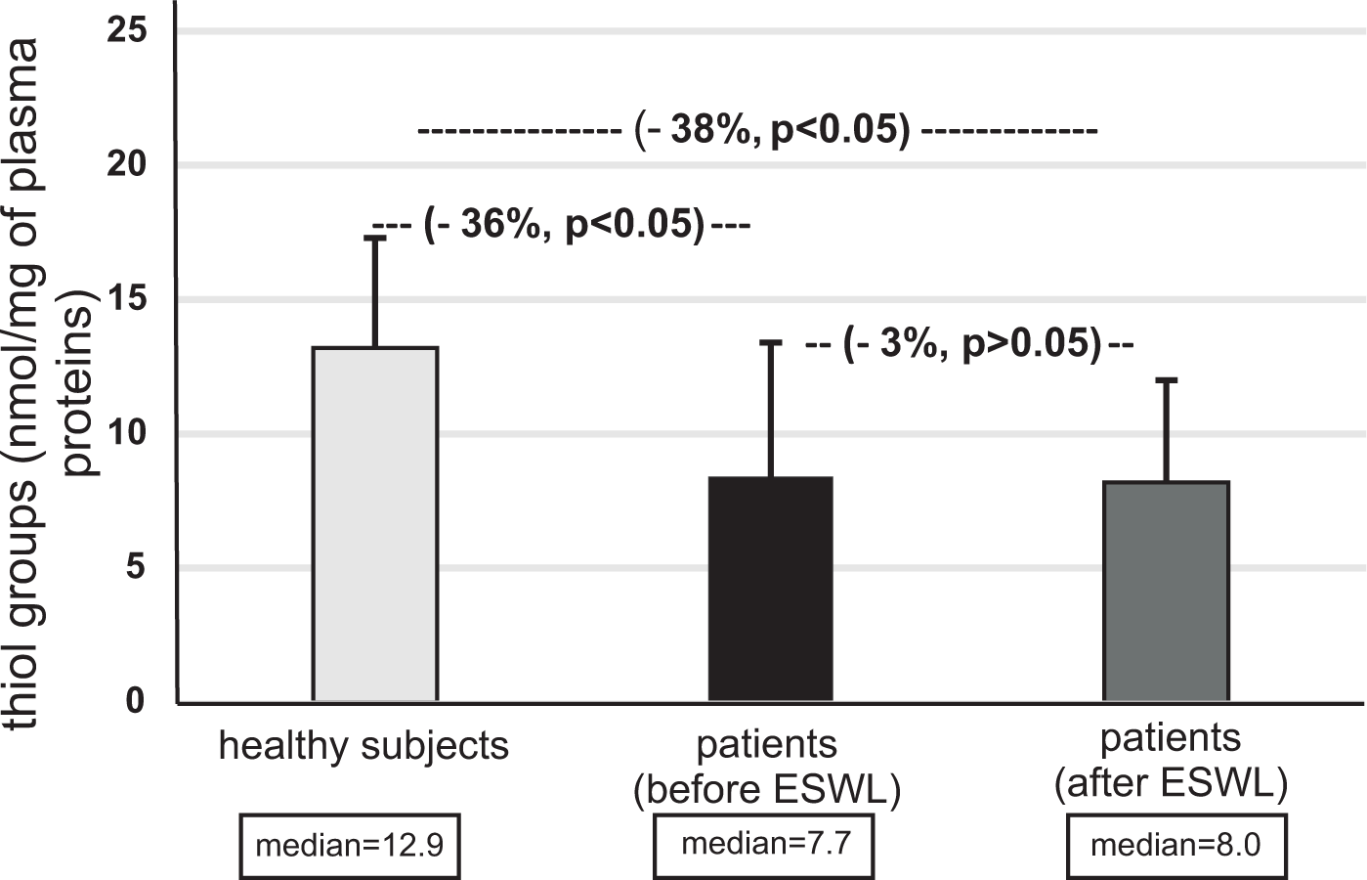
The level of thiol groups in plasma proteins in patients with nephrolithiasis (before and after ESWL), and in control plasma proteins obtained from healthy volunteers. Results are given as means ± SD, and median. The statistical analysis was performed using the Mann-Whitney test.

**Fig 6:**
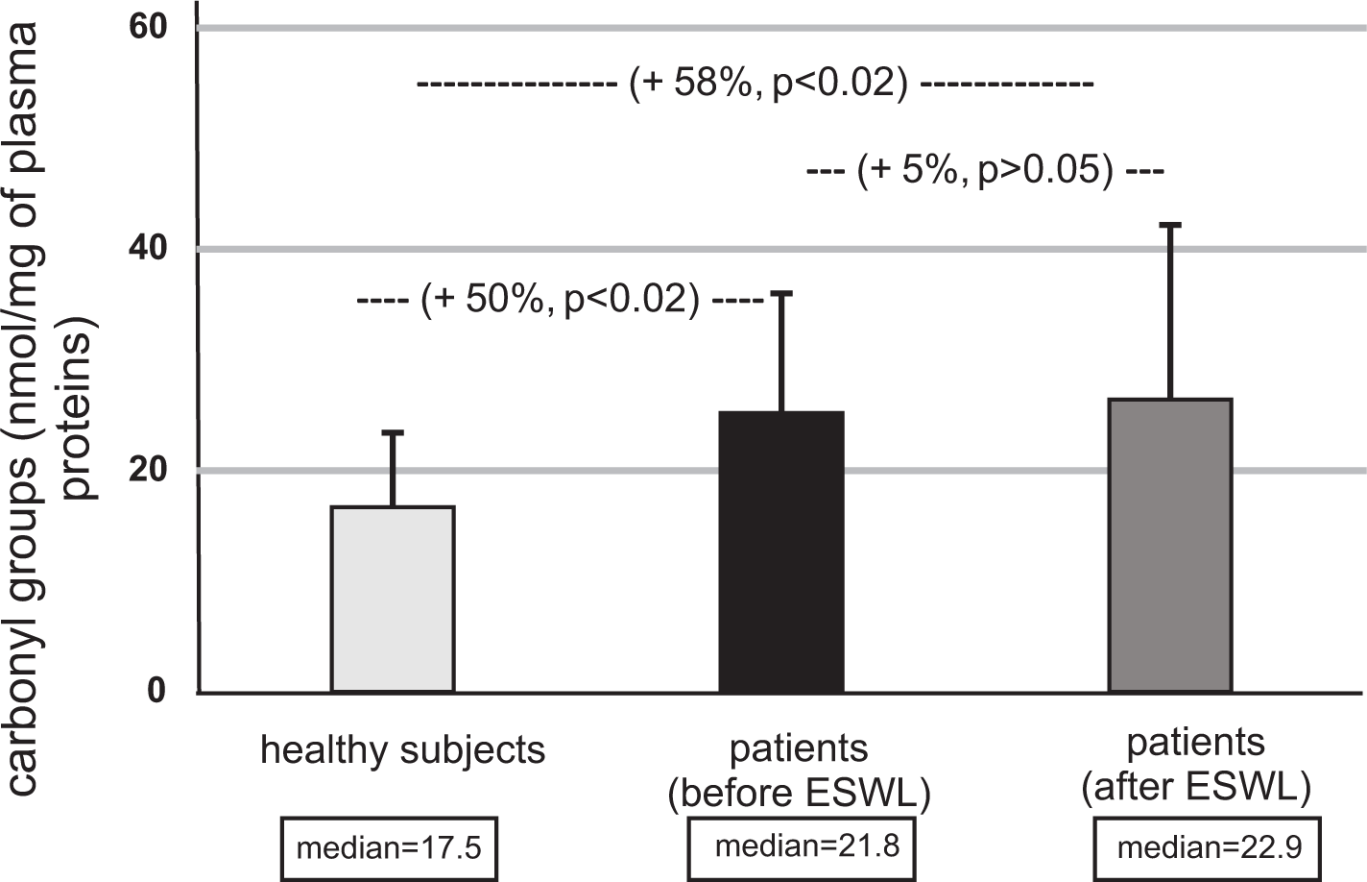
The level of carbonyl groups in plasma proteins in patients with nephrolithiasis (before and after ESWL), and in control plasma proteins obtained from healthy volunteers. Results are given as means ± SD, and median. The statistical analysis was performed using the Mann-Whitney test.

The concentration of thiol groups in plasma proteins from patients (before and after ESWL) was also found to be lower than the concentration of thiol groups in plasma obtained from healthy volunteers (p<0.05) (Fig 5). The concentration of thiol groups in plasma proteins from patients with nephrolithiasis (after ESWL) was also lower than in patients with nephrolithiasis (before ESWL); however, these changes were not statistically significant (p>0.05) (Fig 5). Contrary to the concentration of thiol groups, the carbonylation of plasma proteins from patients with nephrolithiasis (before and after ESWL) was significantly higher than in plasma proteins obtained from healthy volunteers (p<0.02) (Fig 6). However, the level of carbonyl groups in plasma proteins from patients after ESWL was not changed, compared with patients before ESWL (p>0.05) (Fig 6, S3 Table).

## Discussion

A few papers indicate that patients with nephrolithiasis are at increased risk of hemostatic complications, especially increased risk of coagulopathy, following SWL [2,3]. However, the pathogenesis of hemostasis in patients with nephrolithiasis, particularly after forms of treatment such as SWL, is not completely understood. Moreover, oxidative modifications of plasma proteins such as fibrinogen or other coagulation factors may lead to changes in hemostasis [1,13,14].

*In vitro* and *in vivo* experiments demonstrate that increased oxidative stress is correlated with kidney stone development [15–22]. Ma et al. [21] indicate that erythrocyte oxidative stress in patients with calcium oxalate stones correlates with stone size and renal tubular damage. A key novel finding of this study is that patients with nephrolithiasis undergoing ESWL experience an increase in oxidative stress, manifesting as oxidative damage to plasma proteins, as compared to healthy volunteers both before and after ESWL. However, no changes in the oxidative stress were found between patients before ESWL and those who had completed treatment, as measured by the levels of thiol groups and carbonyl groups in plasma proteins. It should be emphasized that the level of oxidative damage to plasma proteins was not found to be dependent on patient age. A recent study by Ceban et al. [22] reports an increased intensity of oxidative stress in patients with renal lithiasis compared to healthy individuals, demonstrated by various biomarkers, including the lowering of thiol groups in proteins. In addition, they note an improvement of these biomarkers after surgical treatment.

Various papers indicate that fibrinogen, a very important protein in the coagulation system, is much more vulnerable to oxidative modifications than others, such as albumin. In addition, oxidized fibrinogen may inhibit thrombin-catalyzed clot formation [1,13,14]. The present study demonstrates not only that oxidative damage is present in the plasma proteins (including fibrinogen) of patients with nephrolithiasis following ESWL, compared to healthy subjects, but also that fibrinogen concentration decreases.

Our present findings indicate a significant increase of another important marker of plasma hemostatic activity, i.e. D-dimer concentration, in nephrolithiasis patients following ESWL, compared to healthy subjects and patients undergoing ESWL. These observations were similar to those of Umekawa et al. [2] and Hughes et al. [3]. Umekawa et al. [2] report a correlation between fibrin degradation products (FDP) and D-dimer concentration following SWL. Interestingly, our present findings do not include any significant changes in PT, TT or APTT in patients with nephrolithiasis, either before or after ESWL. Other authors [3] describe the same observations for PT and APPT in patients after SWL.

Together with the coagulation system and fibrinolysis, blood platelets are a very important element of hemostasis, whose main role is to form mechanical plugs during normal hemostasis. Our study demonstrated a significant decrease in blood platelet count in patients with nephrolithiasis both before and after ESWL. Our findings compliment those of Dedej et al. [4] and Hughes et al. [3]. Hughes et al. [3] report a decreased blood platelet count at 30, 120 and 240 min following SWL. They suggest that the changes of blood platelet count and fibrinogen observed when taking readings 30–240 min after SWL may be due to their redistribution in the body following renal trauma caused by SWL: blood platelet counts and fibrinogen will be consequentially increased in the kidney immediately after SWL, and decreased at the peripheral blood. It is also possible that a similar process exists in our present experimental model. Moreover, considering the data presented in this study, it is likely that the oxidative stress present in patients with nephrolithiasis, both before and after ESWL, may induce changes of hemostasis in these patients. Results of Moyes et al. [5] also demonstrated significant changes in haematology (i.e. the decrease of blood platelet count and fibrinogen concentration) and in biochemistry parameters, including the increase of APTT, following Flexible Ureterorenoscopy.

Further experiments based on larger groups of patients will be needed to determine the effect of oxidative modifications of plasma proteins on hemostatic abnormalities observed in patients with nephrolithiasis before and after ESWL. In addition, a deeper analysis of specific parameters of hemostasis, i.e. fibrinogen, D-dimer and blood platelet count, may provide valuable data on the hemostatic response following ESWL, and may decrease the risk of coagulopathy in patients with nephrolithiasis after ESWL. By having a better understanding of the influence of ESWL on hemostasis, this could lead to modifying patient care for those patients at increased risk of bleeding.

## Supporting information

S1 Table The activated partial thromboplastin time (APTT), prothrombin time (PT), and thrombin time (TT) of plasma in patients with nephrolithiasis (before and after ESWL), and in control plasma obtained from healthy volunteers. (TIF)

S2 Table The level of fibrinogen and D-dimer in plasma and the level of blood platelets in patients with nephrolithiasis (before and after ESWL), and in control obtained from healthy volunteers. (TIF)

S3 Table The level of thiol groups and the level of carbonyl groups in plasma proteins in patients with nephrolithiasis (before and after ESWL), and in control plasma proteins obtained from healthy volunteers. (TIF)

## Abbreviations

APTT: activated partial thromboplastin time of plasma
ESWL: extracorporeal shock wave lithotripsy
PT: prothrombin time of plasma
SWL: shock wave lithotripsy
TT: thrombin time of plasma
