# Supplementary figures and images for: Evaluation of hemostasis parameters and the role of the oxidative damage to plasma proteins in the modulation of hemostasis in patients with nephrolithiasis before and after extracorporeal shock wave lithotripsy
Source: PLoS One. 2017 Oct 2;12(10):e0185157. doi: 10.1371/journal.pone.0185157 (PMC5624585; doi:10.1371/journal.pone.0185157)

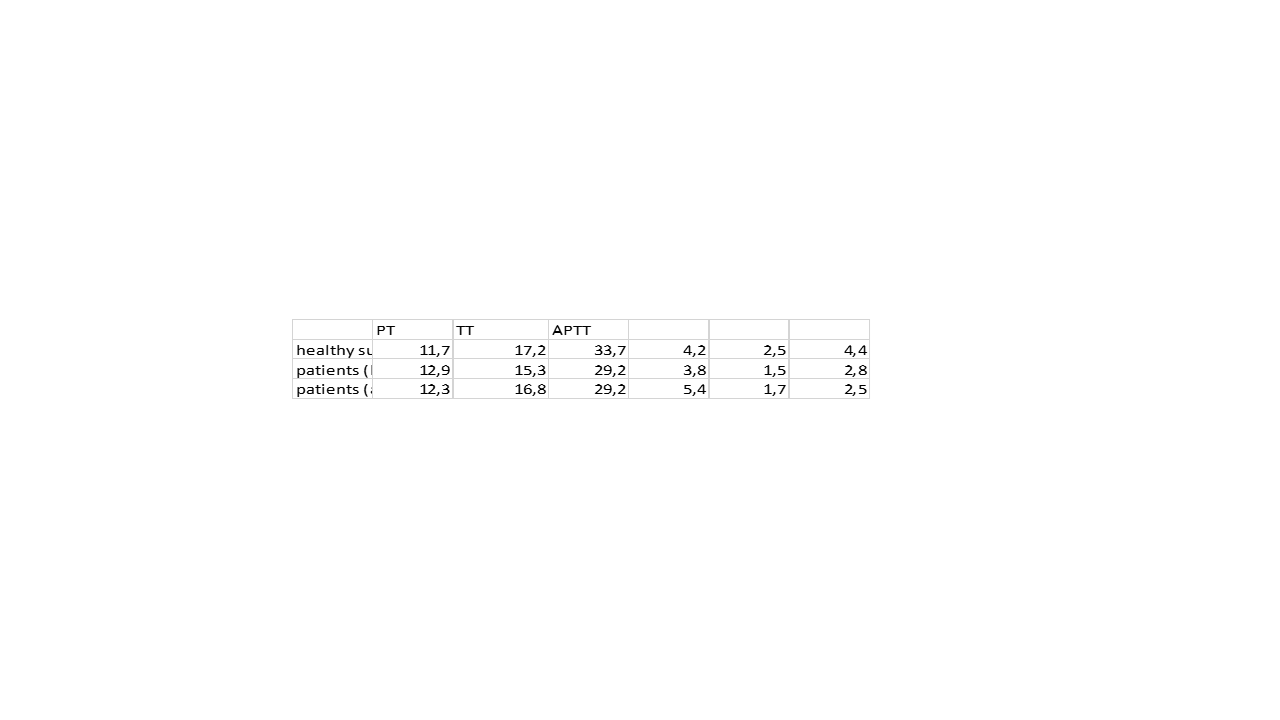

Supplement: S1 Table — (TIF) [file pone.0185157.s001.TIF]

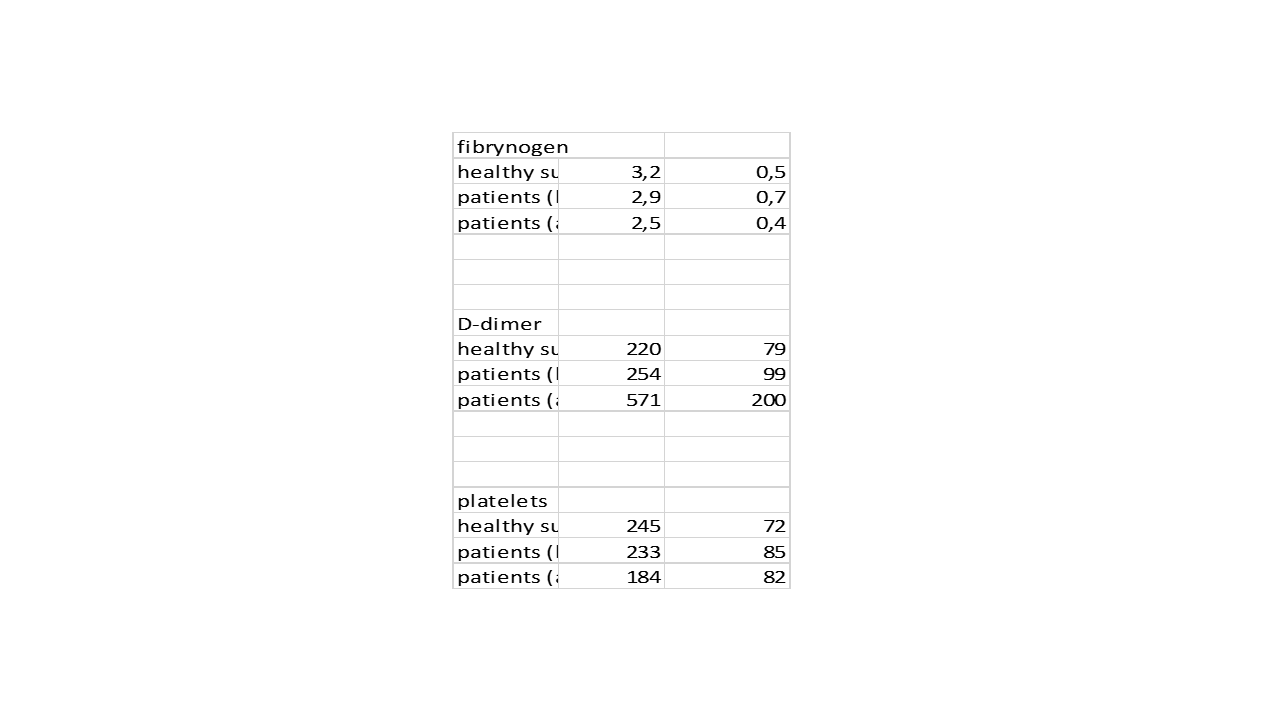

Supplement: S2 Table — (TIF) [file pone.0185157.s002.TIF]

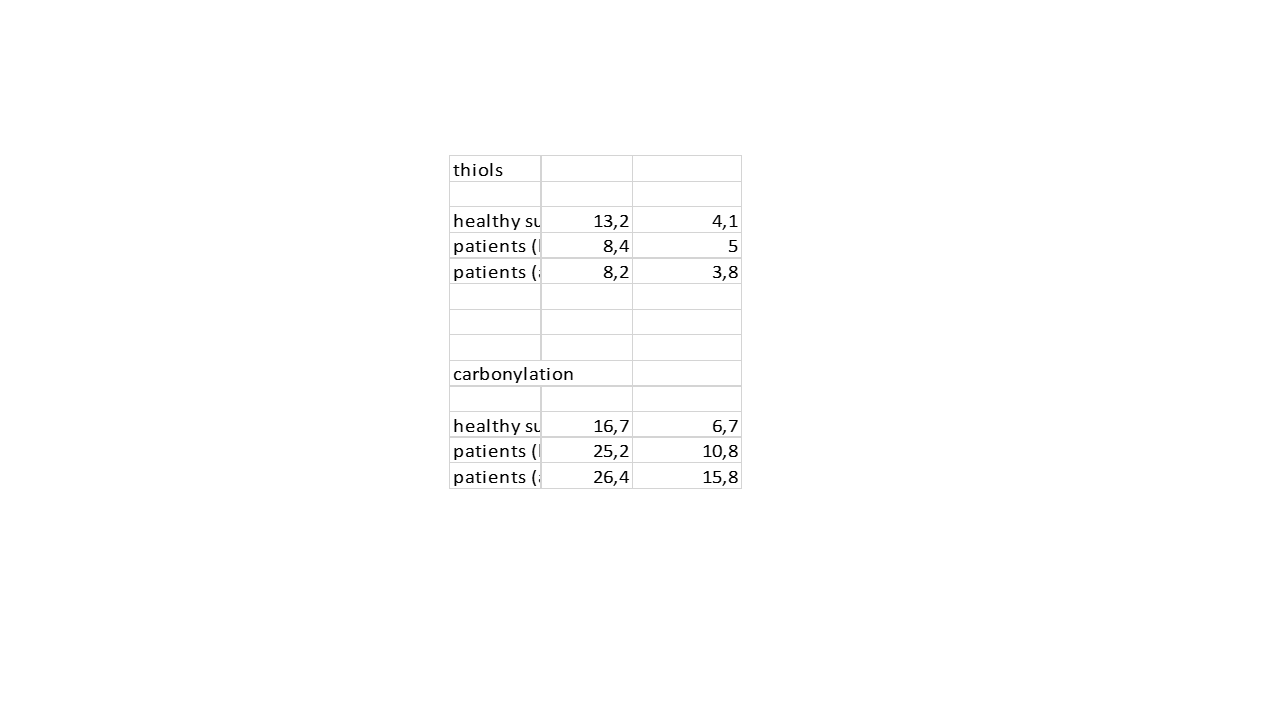

Supplement: S3 Table — (TIF) [file pone.0185157.s003.TIF]
